# Remarkably reproducible psychological (memory) phenomena in the classroom: some evidence for generality from small-*N* research

**DOI:** 10.1186/s40359-022-00982-7

**Published:** 2022-11-22

**Authors:** Abdulrazaq A. Imam

**Affiliations:** Department of Psychology, John Carroll University, 1 John Carroll Blvd, University Heights, OH 44118 USA

**Keywords:** Reproducibility, Replication, NHST, Memory, Experimental design, Small-*N* designs, History of psychology

## Abstract

**Background:**

Mainstream psychology is experiencing a crisis of confidence. Many of the methodological solutions offered in response have focused largely on statistical alternatives to null hypothesis statistical testing, ignoring nonstatistical remedies that are readily available within psychology; namely, use of small-*N* designs. In fact, many classic memory studies that have passed the test of replicability used them. That methodological legacy warranted a retrospective look at nonexperimental data to explore the generality of the reported effects.

**Method:**

Various classroom demonstrations were conducted over multiple semesters in introductory psychology courses with typical, mostly freshman students from a predominantly white private Catholic university in the US Midwest based on classic memory experiments on immediate memory span, chunking, and depth of processing.

**Results:**

Students tended to remember 7 ± 2 digits, remembered more digits of **π** following an attached meaningful story, and remembered more words after elaborative rehearsal than after maintenance rehearsal. These results amount to replications under uncontrolled classroom environments of the classic experiments originally conducted largely outside of null hypothesis statistical testing frameworks.

**Conclusions:**

In light of the ongoing replication crisis in psychology, the results are remarkable and noteworthy, validating these historically important psychological findings. They are testament to the reliability of reproducible effects as the hallmark of empirical findings in science and suggest an alternative approach to commonly proffered solutions to the replication crisis.

## Background

“…a reproducible finding may not necessarily be true; however, a finding that fails reproduction or replication under identical conditions is most likely false. An additional factor operative in social sciences is the subjects’ beliefs and information available to them, which dilutes the concept of objective truth and exacerbates the epistemological divergence between reproducibility and validity of scientific results.” [1]

“It is possible that different psychological science subfields have different priors and different biases, so it would not be surprising if the proportion of unchallenged fallacies varies considerably across subfields (e.g., from 30 to 95%). Then, the remaining 66–1%, respectively, would be unconfirmed genuine discoveries. In all, the overall credibility of psychological science at the moment may be in serious trouble.” [2]

There is wide acknowledgement of a twin crisis in psychology and beyond (e.g., [3], see [2]), namely, widespread questionable research practices (QRPs) and failures to replicate or reproduce important findings in psychology such as in precognition [4, 5] and priming [6–9]. It appears the pervasive adoption of inferential statistics in the form of null hypothesis statistical testing (NHST) is a contributing factor (see [10]) even as the second of these crises manifests to varying degrees across disciplines (e.g., [1, 11–14], see [15]). In psychology, the much proclaimed replication failures may have been, in part, a byproduct of the first, in that QRPs naturally flowed out of the almost blanket adoption of NHST as a primary means of analyzing and evaluating data (see [1, 16]). Almost blanket because some areas of psychology, particularly behavioral psychology, had a wholly different approach to data analyses and evaluation. According to Smith and Little, there are pockets of use of this approach in cognitive psychology as well (see [17, 18]). As such, psychology probably is unique in effectively having more than one research tradition. Notwithstanding, the solutions that have been adopted to deal with these crises have tended to focus only on one of them, almost as if there is just one such tradition in practice. Solutions surrounding the adoption of the “new statistics” [19] including advocacy for different replication efforts [10, 20] have been tailored narrowly to address the ubiquity of NHST and its impacts on psychological research (see [21]). The two statistical alternatives typically offered up for consideration, namely, the frequentist “new statistics” (e.g., [19, 22]) and Bayesian statistics (e.g., [23, 24]), actually belong in one tradition within psychology (see [25]) as elaborated below.

The import of the opening quotations to this section is that, on the one hand, psychology in general, like other social sciences, uniquely deals in human phenomena that necessarily evolve an epistemological gap between replications and validity of its findings. On the other hand, although specific areas of psychology vary in their respective production of false positives, the net result is the credibility crisis that befalls the whole discipline. Distinctions we make on some topics in psychology may be arbitrary and capricious. Such is the case with memory, which is ordinarily considered cognitive at large. This paper argues that the methodologies deployed to study the phenomenon in classical times would not be considered appropriate for its study today largely because it happens to be cognitive in today’s terms. As such, standard mainstream methodologies involving group designs would apply typically. Current subfield differentiations (e.g., between cognitive vs. behavioral), however, blur the historical and epistemological significance of the nexus between replicability and methodology, on the one hand, and between methodology and validity, on the other. Certain aspects of memory have benefited historically from making contacts with different methodologies that afford an evaluation of their validity. The first part of the following review argues that indeed there is yet another largely neglected option in the ongoing remedial efforts that is worthy of serious consideration (see [18, 26, 27]) in addition to those currently on offer for dealing with the aforementioned crises. The final part of the review situates the memory phenomena reported here in the context of the historical reality of a dual research tradition in psychology.

### Two research traditions in psychology

Broadly speaking, psychology has two research traditions historically. One that is predominant today involves large-*N* group designs. In this approach, researchers tend to begin with stated hypotheses tested using appropriate experimental designs informed by specific statistical considerations and assumptions, which may or may not be fulfilled in practice, followed by data analyses and interpretations deployed to answer them. In preponderance of the times, the latter usually involves deploying NHST, which has been the subject of numerous and intensive criticisms for various pitfalls (see [28–33]). Although the goal of such research is to achieve extrapolation from the sample to the population, often the population is not well defined and there is substantial dependence on largely undergraduate convenience samples (see [18, 25, 34]). Use of convenience samples represents a departure from untenable random-sampling assumptions that statistic analyses rely on to justify the conclusions reached about observed effects [1]. Hanin made the case, for example, that “… (a) arbitrarily small deviations from the random sampling assumption can have arbitrarily large effects on the outcomes of statistical analyses, (b) the commonly occurring observations with random sample size may violate the Law of Large Numbers (LLN, which make them unsuitable for conventional statistical inference…” [1], p. 2). In these and many other ways, one could fault psychologists for poorly using the best statistical tools (see also [35], p. 221).

Historically, the NHST approach represents a hybrid of two distinct statistical positions in psychology, namely, Fisher’s statistical significance testing (SST) and Neyman–Pearson’s statistical hypothesis testing (SHT; [36]). There were fundamental differences between the two, some of which are irreconcilable, but the hybridization occurred nevertheless (see, e.g., [37]), usually without a hint of the history in statistics or methodology textbooks [1]. The outcome has been a terribly flawed process of interpretation of psychological research findings [30, 32, 38–40]. One major flaw is the false conception of the *p* value as an index of confidence in the results; another is the seriously mistaken belief by many that it represents replicability of the results [33, 37], see also [41]. Perhaps partly due to the latter erroneous replicability posture on the meaning of the venerated *p* value in extant psychology, there have been aforementioned failures in replication practices and reproducibility of important psychological findings (e.g., [5, 6]) resulting in new efforts at promoting replications (see [20, 42]), on one hand. On the other hand, NHST alternatives such as the new statistics recommending the use and reporting of effect sizes, confidence intervals, and meta-analyses [19, 22, 43–47] and Bayesian statistics [23, 24, 48] have been proffered. As Smith and Little [18] aptly observed, there has been an inadvertent demand for larger and larger samples in various journals as a matter of policy because of these efforts, to the detriment of the science we seek to advance particularly given the exemplary beneficial scientific features [45] of the alterative.

The alternative tradition has a long history in psychology, antedating the rise and eventual dominance of NHST in psychological research, namely, small-*N* experimental designs that some describe as *N* = 1 or *N*-of-1 [25, 38, 49–51]. Deployed frequently in psychophysics [52–57], it has roots in Fechner’s earliest works (see [54, 58]). The approach typically does not require a reliance on inferential statistics for evaluating data primarily because of its heavy reliance on experimental rather than the statistical control that is intrinsic to group designs ([51, 59–61], see [62], for historical usage). Additionally, it has the unique characteristic of inherently requiring replications as a matter of course (see [17, 18, 27]). In this tradition, research may begin with a formal hypothesis not driven by statistical considerations (see [45]) or with an informal hunch about some functional relationship between independent and dependent variables. What drives the outcome is the rigor of experimental control used in demonstrating such functional relationships for the same subjects by repeated exposure to various conditions (intrasubject replication), between different subjects exposed to similar conditions (intersubject replication), or across settings, situations, species, etc. In so doing, it establishes not only a strong internal validity but also generality of the effects [18, 51, 63]. Primarily, evaluation of data is conducted typically with graphical depictions of patterns of change in the dependent variable of interest (see [61, 64]), mostly relying on visual inspection of the data.

Although often credited with the founding of psychophysics [56, 65], which also has been traditionally reliant on extensive studies of only few subjects, ironically, Fechner is also credited with introducing “statistical methods” to psychology in terms of what Stigler described as “probability-based modeling and inference” [18, 58]) tends to rely mostly on the use of large-*N* group designs with their attendant complexities, whereas behavioral psychology tends to rely mostly on the small-*N* experimental designs [35]. The two areas of psychology tend to approach their subject matters reliably from different vantage points. Conceptually, for example, the subject of memory is characterized alternatively as *remembering* in behavior analysis to reflect long-standing recognition of the phenomenon as an action event, as opposed to a hypothetical construct (e.g., [66], see also [67], White and Wixted [56]). Now, because memory is construed typically as a cognitive phenomenon, one might expect, for sure, from a contemporary standpoint, that it would be studied using the standard cognitive methodology relying on large-*N* group designs.

The focus of the present study is the reliability of reproducing studies of memory presumably conducted from a cognitive perspective that did not historically rely on large-*N* group designs for the most part. In doing so, one hopes that the current crises on issues of replications and reproducibility of psychological phenomena [20, 68, 69] would illuminate the methodological issues involved. Ebbinghaus’ study of memory was prominent in Dukes’ [50] enumeration of important psychological reports that used *N* = 1 research. The reliability of reproducible effects is the hallmark of empirical findings in science after all. Achieving field replications in Huffmeier et al.’s [70] replication typology provides such reliability for the memory phenomena reported here. As highlighted further below, Ebbinghaus’ memory work has had a long history of successful replicability. To be sure, there have been other important discoveries in psychology that derived from studies that did not rely on inferential statistics commonly used in large-*N* group designs [71]. The classic memory studies reviewed here appear to belong in the same caliber of studies. They cover three different important topics on memory: (1) immediate memory span, (2) chunking, and (3) levels of processing.

### Classic memory studies

“Psychological knowledge is not acquired *a priori* – we cannot know in advance what will emerge as reliable findings without replicating initial findings.” [72

Findings from classic memory experiments on immediate memory span (e.g., [73–75], see [76]), chunking [74], and level of processing (e.g., [77]), have had long-standing impact on our understanding of memory processes in psychology. Fifteen of the 20 articles (75%) cited in Miller’s [74] review that culminated in the magical number seven were published in the 1950s, only three (15%) from the 1930s, and one each from 1945 and 1904 (10%). But for the 1904 citation, the seminal works of Guilford and Dallenbach [73] and Oberly [75] on immediate memory spans that informed Miller’s review antedated all of these works. What is noteworthy about the two earliest works is that they studied memory processes using experimental designs devoid of statistical inferences. Guilford and Dallenbach’s study, for example, was “an intensive study upon a few Ss, and extensive study upon a large class” [73, 75] worked with seven participants presented with 2–14 digits whose memory spans ranged from 6 to 14. Oberly’s extensive study involved 100 participants presented with 4–12 digits either randomly or in sequence yielding memory spans of 8.9 each. Notably, again, Oberly did not deploy inferential statistics; indeed, the remaining narrative and discussions by Oberly following the presentation of the group data focused largely on the verbal reports of the seven individual participants.

On the topic of immediate memory spans, Miller’s reviews of absolute judgement of unidimensional and multidimensional stimuli concluded, that “[t]here is a clear and definite limit to the accuracy with which we can identify absolutely the magnitude of a unidimensional stimulus variable,” which he specified to be “in the neighborhood of seven” [74*chunks*, which he argued could be circumvented by processes involving “recoding” in which we may construct “larger and larger chunks, each chunk containing more information than before” [73, 74] and Oberly [75], Miller’s review included some works that did not employ inferential statistics either. Pollack [78], for example, studied verbal learning by 25 participants and reported their mean data, without any apparent appeal to inferential statistics for interpretation of the outcomes. Carmichael et al. [79] was another one of the papers reviewed to support the influence of naming on visual perception. In their study, they presented visual images with two lists of labels for various objects to 48 and 38 experimental participants, respectively, and 9 control participants who got no names. The analyses and interpretation of the results involved no statistical treatments at all. Thus, even the studies that employed large number of participants did not resort to inferential statistics to make sense of the data.

As Murray’s historical analyses of the influence of nineteenth century memory research concluded, modern memory research topics such as level of processing [77] have some connection to nineteenth century work on memory. Much of the research on memory from the era were notably of the small-*N* variety in the tradition of Ebbinghaus’s groundbreaking self-experimentation with nonsense syllables (e.g., [80]) as were those from early twentieth century [81–84]. Even modern replications of Ebbinghaus have stayed true to the tradition (e.g., [85, 86]. Kirkpatrick [87] conducted memory experiments with large numbers of students, but still did not rely on inferential statistics for interpretation of the results. By the time of the publication of Miller’s paper in 1956, the use and reporting of group designs and inferential reporting of *p* values in psychological research had just reached its peak [25, 62, 88] having virtually replaced *critical ratios* and *probable errors* that were prevalent when the Guilford and Dallenbach [73] and Oberly [75] papers had appeared. Predictably, then, much of the work reviewed by Craik and Lockhart [77] derived from mainstream psychology research that emphasized reporting of NHST. In their paper, Craik and Lockhart advocated meaningful, deeper processing as an aid to retention of information. Moscovitch and Craik [89] provided some empirical evidence in support of the depth of processing view of memory. They reported three experiments demonstrating better recall with meaningful sentences than with rhymes using large number of participants and NHST analyses and interpretation of the data. In the same vain, four other studies reanalyzed in light of the depth of processing notion all used a large number of participants and inferential statistics to report their findings [90–94]. The exclusive reliance of NHST in relevant level of processing research then reflects its widespread adoption in mainstream psychology.

Recent experimental replications of Ebbinghaus’ memory experiments and the use of savings have variously stayed true to his methods (e.g., Murre and Dros [85]) by using small number of participants, using syllables as stimuli, and using the method of savings as the primary dependent variable. Murre and Dros provide the most modern replication of Ebbinghaus’s memory experiments that stayed close to his approach. Even they, however, succumbed to the analytical zeitgeist by occasionally reporting NHST in their data analyses, perhaps reflecting Ebbinghaus’ tendencies for methodological eclecticism [95]. Their results though, notably, confirmed Ebbinghaus’, supporting the robustness of the generality of the memory phenomena explored. The versatility of the subject matter combined with the rigor of the methodology used in the original classic studies makes it conducive to examine the recent contemporary problems of replication and reproducibility of findings afflicting psychological science. The replicability of the Ebbinghaus memory phenomena (Murre and Dros 2017) illustrates the point. The classic memory studies of immediate memory span, chunking, and levels of processing offer additional lines of evidence for demonstrating generality of effects reported using methods other than those widely employed in mainstream psychology today. Collectively, they have withstood the test of reproducibility having been reliably reproduced well within experimental laboratory preparations. The latter classic cases, the subjects of the present report, particularly provide an opportunity to explore the extent of the generality supported by their largely small-*N* experimental roots. The opportunity is not one of a prospective study of these memory phenomena, however.

Many introductory textbooks provide demonstration activities (e.g., [96]) on these phenomena for the classroom. Three memory exercises on immediate memory span, chunking **π,** and depth of processing comprised the retrospective examination of results from classroom demonstration activities conducted between 2013 and 2019 in various introduction to psychology courses including special sections on social justice. The activities reflect specific attributes of the classic studies discussed above, all being cognitive processes that would not be considered appropriately studied with the original methodologies in today’s psychology. They also shared in common that completing these exercises involved quantitative data collected at the time of the demonstrations. Classroom demonstrations, of course, occur in environments unlike the laboratories that produced the original experiments establishing these phenomena. If under such uncontrolled environments they succeed in reproducing the expected effects, they further attest to the robustness of the original findings, provide ecological extensions of those findings, and present interesting implications for our understanding of the experimental design and analyses deployed for their original empirical reporting in contemporary context. What follows describes the procedures used to collect the retrospective data in various classrooms.

## Method

Undergraduate students enrolled in introductory psychology courses over multiple semesters and across many years from 2013 to 2019 participated in classroom memory demonstrations. They were typical, mostly freshman students from a predominantly white private Catholic university in the US Midwest. Table 1 shows the activities for which data were collected including those from introductory psychology classes with social justice themes. Of the three activities, namely, immediate memory span, chunking **π**, and depth of processing, data on immediate memory span was limited to the fall of 2017 through fall of 2019. Each activity was implemented using the instructions provided in the instructor’s materials (IM) that accompanied Bernstein’s [96] introductory psychology textbook along with the materials and display items for each demonstration:

**Table 1:** Years (and semesters) of data collection on immediate memory span (IMS), chunking **π** (Chunking), and depth of processing (DoP) activities in introductory psychology classes

Activity	Year (Semester)													
	2013		2014		2015		2016		2017		2018		2019	
	Spring	Fall	Spring	Fall	Spring	Fall	Spring	Fall	Spring	Fall	Spring	Fall	Spring	Fall
IMS										X*	X*	X*	X^†‡^	X*^‡^
Chunking		X	X		X				X*	X*	X*	X	X*^‡^	X*^‡^
DoP		X	X	X	X			X	X*	X*	X*	X	X^†‡^	X*^‡^

* = 2 sections; ^†^ = 3 sections; ^‡^PS100 (Social Justice) sections included

### Immediate memory span (IMS) exercise

The immediate memory span exercise was Activity #1 on short-term memory of Supplement 8.10 in the IM that accompanied Bernstein [96]. The stimuli were 10 series of digits starting with three digits and ending with 12 digits, each series increasing by one digit.

Students saw the numbers displayed one digit-at-a-time with increasingly longer number of digits in each subsequent series. At the end of each series, following a very brief pause, they wrote down the digits in sequence. After all the series have been presented individually, students saw all the digits in all series at once to check against their written series, and then determined their IMS from the one preceding the series with their first error. Headcount of their span followed, with a discussion of 7 ± 2 capacity of short-term memory.

### Chunking π exercise

The chunking exercise appeared in Supplement 8.11 of Bernstein’s [96] IM. According to the instructions, students saw 20 digits of **π** on the screen to examine briefly. They then wrote down as many digits of **π** they could remember after a distraction task. Headcount of students remembering digits from 20 to 1 followed (Before). The digits then were displayed, grouped to accompany a story narrated to the class. Following the distraction task again, students wrote down as many digits of **π** they could remember. Another headcount for digits recalled followed (After), with display of tallies and discussion of chunking and the roles of meaningful processing.

### Depth of processing (DoP) exercise

The depth of processing exercise provided implementation instructions and the accompanying task instructions for students in Supplement 8.14 to illustrate the level of processing model of memory. The exercise began by dividing the class into two groups, A and B. One group received instructions to count vowels (maintenance rehearsal) and the other to find usefulness on an island (elaborative rehearsal) in words read aloud to the class. The respective instructions were displayed on the screen. When Group A received its instructions, Group B had eyes closed and vice versa. The list of words included 22 words ranging from umbrella to bottle. Following a distraction task that lasted about 30 s during which students wrote down their name, address, phone number, major, and social security number, they wrote as many words from the list as they could remember. Headcount of how many words remembered by each group and a subsequent discussion of levels of processing then followed.

## Results

The data reported were all count data collected by show of hand in the classroom. If the memory span activity is successful, students would remember mostly between 5 and 9 items, inclusive, as predicted based on the classic studies on the topic (see [74]). Success in the chunking exercise entails students remembering more digits of **π** *after* the meaningful story than *before* it. Because the putative data derived from head counts in the present study, recalled items could not be matched before and after for each student as would be customary in a laboratory version of the study using a small-*N* design. Chunking predicts remembering more digits of **π** due to recoding into larger units [74], in the present case, students should remember more digits, accordingly. Finally, success in the DoP activity is reflected in the students who received instructions for maintenance rehearsal remembering less than those instructed for elaborative rehearsal in accord with level of processing theory of memory [77].

**Fig. 1:**
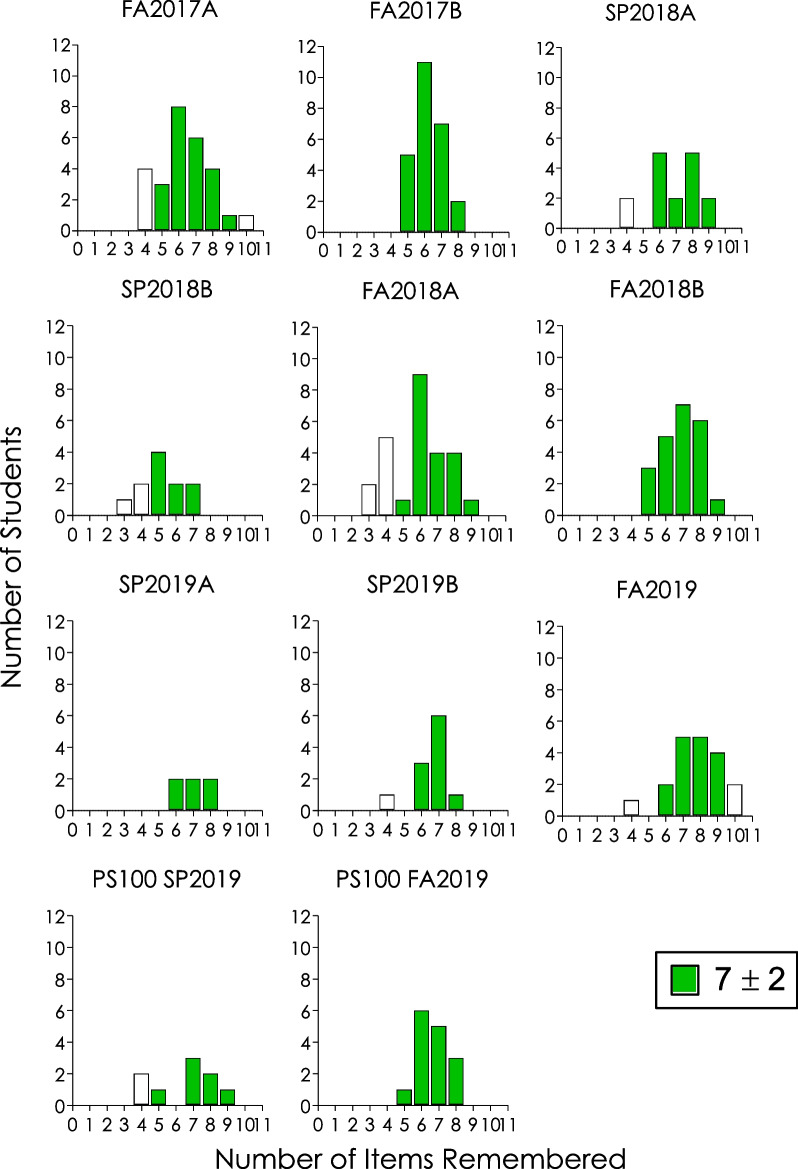
Number of students who remembered a given number of items in an immediate memory span demonstration exercise in introductory psychology courses across 11 sections in 5 semesters from 2017 to 2019. Letters A and B represent different sections of the course in the same semester and green bars reflect data within 7 ± 2

Figure 1 presents data from 11 sections during five semesters starting from 2017 through 2019 on immediate memory span. Each graph presents a semester’s data from each section of introductory psychology including the last two showing those of the special sections on social justice (PS100). Figure 1 shows that most students remembered items more within the 7 ± 2 span in each semester indicated by the colored bars. Whereas most sections, 7 of 11 sections (64%), recorded students below the 7 ± 2 span, only two (FA 2017A and FA 2019) did so above the span representing 18%. Incidentally, the two sections recording students above the span were among the sections with students below the span; FA2017A recorded 4 below and 1 above, whereas FA2019 recorded 1 below and 2 above.

**Fig. 2:**
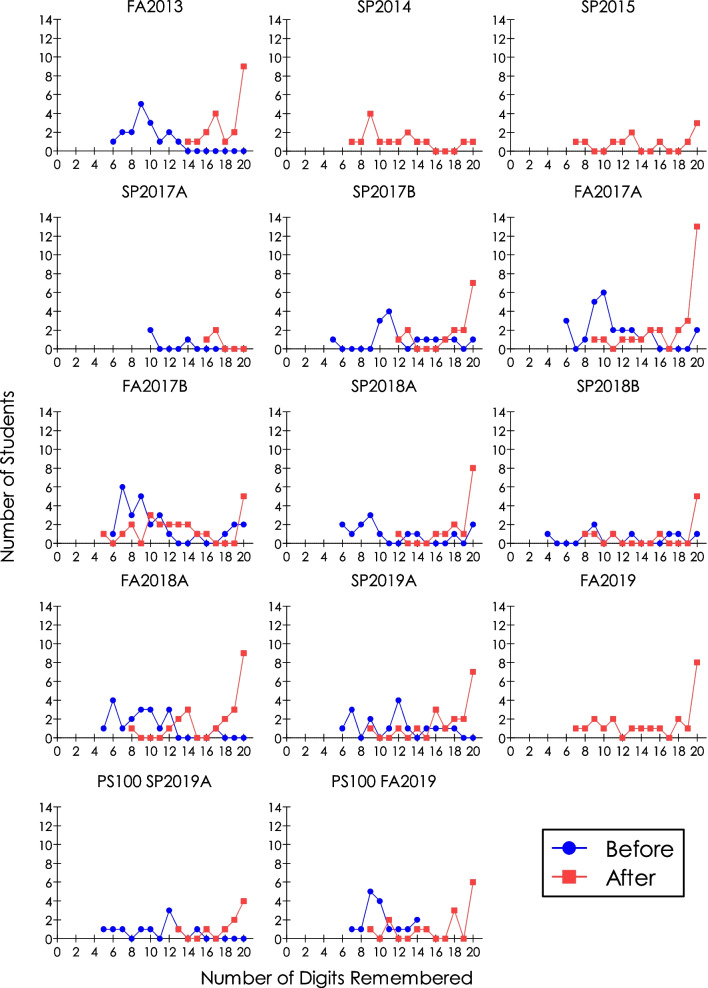
Number of students who remembered digits of **π** as a function of number of digits remembered before (blue circles) and after (red squares) hearing an arbitrary story containing digits of **π** to the 20th digit in introductory psychology courses across 14 sections in 9 semesters from 2013 to 2019. Letters A and B represent different sections of the course in the same semester

Figure 2 presents the *before* and *after* counts of students who remembered **π** to the 20th digit across nine semesters from 2013 through 2019 in 14 sections. Data *before* the story were not available for three semesters, spring of 2014 and 2015, as well as fall of 2019. As such, adequate comparisons are possible for only 11 of 14 sections. The figure shows that in all semesters where comparisons are possible, students tended to remember more digits of **π** following the narrated, meaningful (albeit arbitrary) story that accompanied the digits (shown in red in the figure) than *before* the story (shown in blue). Visual inspection of the graphs reveals the effect in two different ways. First, there were higher peaks in the number of students remembering the digits of **π** *after* (peaks were at 20th digit, except for SP2014 at 7th digit and SP2017A at 17th digit) than *before* (peaks were between the 7th and 12th digits across the sections) the story. That is, in 12 of 14 cases (86%), more students recalled the 20 digits (indicating later peaks) *after* the meaningful story, in contrast to before it. Second, there were rightward shifts in the overall number of students remembering **π** *after* the story compared to *before* it. In the three sections without the *before* data, students tended to remember more digits comparable to those of the other 11 sections with *before* data.

**Fig. 3:**
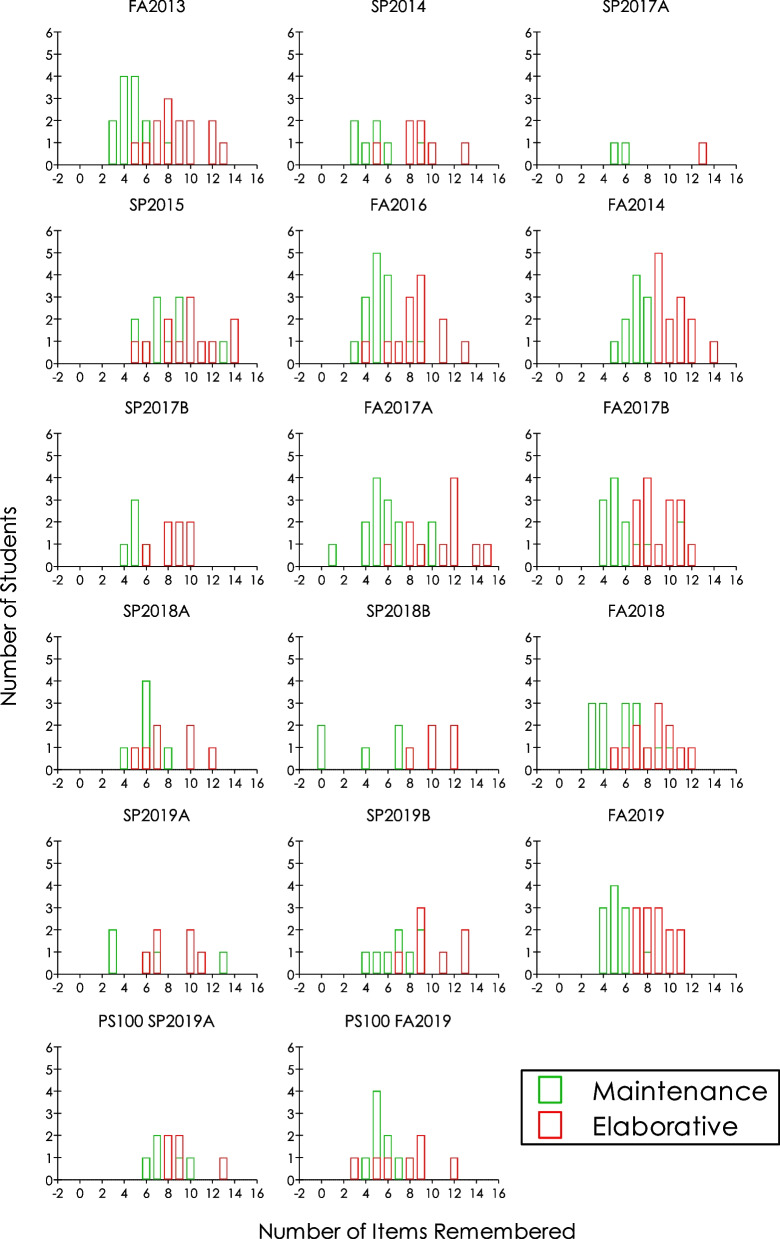
Number of students remembering items as a function of number of items remembered following maintenance (green bars) or elaborative (red bars) rehearsals in a depth of processing demonstration exercise in introductory psychology courses across 17 sections in 14 semesters from 2013 to 2019. Letters A and B represent different sections of the course in the same semester

Finally, Fig. 3 presents the number of students who remembered list items following a maintenance rehearsal task compared to an elaborative rehearsal task. The data presented were from 14 semesters starting fall of 2013 through 2019 in 17 sections, each graph representing a section’s data for each semester. Visual inspection of the figure shows that, in each section, students remembered more words when instructed to find how the list items could be useful to them when stranded on an island (elaborative rehearsal; in red) than to count the vowels in the words read to them (maintenance rehearsal; in green). The rightward shifts in the student distributions with elaborative rehearsal is indicative of this effect; there was a lone student in the SP2019A section who remembered more with maintenance rehearsal than students who used elaborative rehearsal. That is, the effect occurred in 94% of the sections.

Each set of results from the memory span, chunking, and depth of processing showed discernible patterns across the semesters that generally were outcomes consistent with the findings of the original memory experiments in psychology. In each case, the graphical presentations sufficiently depicted the various effects primarily by visual inspection and therefore required no inferential statistical analysis to understand the effects. In experimental data, we seek regularities, in exception to irregularities [51]. In each activity in the present study, amidst any variability in counts, the pertinent data displayed outcomes in line with the previous classic studies, most students remembered 5–9 items, students remembered more digits of **π** *after* than *before* the meaningful story, and students remembered more with deep processing than with superficial processing.

## Discussion

### Methodological legacy

“It is possible that, in several fields of psychological science, the current dominant paradigm when replication is attempted is that of perpetuated fallacies. Replication efforts, rare as they are, are done primarily by the same investigators who propose the original discoveries.” [2

This is not the case in these memory phenomena, even under uncontrolled environments. First, in determining the immediate memory span, students remembered items within the magical 7 ± 2 range in each semester. Each section from each semester represented an independent replication. As such, there were 11 of 11 (100% of the sections) successful replications of this effect; success signified by the number of students remembering 5–9 items (see Fig. 2). Although 64% of the cases recorded occasional spans below Miller’s [74] minimum of five, they did not rise to the same level of evidensory support for a memory span of four suggested by Cowan [97, 98] (cf. [62, 99–102]). Second, wherever possible (in 79% of the sections), there were rightward shifts in the number of students remembering more digits of **π** *after* compared to *before* the meaningful story was attached to **π** digits; in two of the three sections without the *before* data, the rightward shifts peaked at the 20th digit. Altogether, then, there were 14 rightward shifts and peaks at the 20th digit in students recall of **π**; that represents 14 replications of the positive effects of attaching meaningful story to the 20 digits of **π**. Finally, for every semester, students remembered more following elaborative than following maintenance rehearsal. There were 17 sections showing the effect, representing 17 successful replications (i.e., 100%).

These results collectively are indicative of the robustness of the respective phenomena demonstrated; they established the validity of the outcomes of historically important psychological findings on memory span, chunking, and depth of processing [73–75, 77], replicated under uncontrolled classroom environments. They each were discernible by visual inspection without statistical inference. Most students remembered 5–9 items (Fig. 1), students remembered more digits of **π** after hearing a linked story (Fig. 2), and tended to remember better with meaningful processing than with superficial processing (Fig. 3). Note that the variability in students remembering **π** digits is present both before *and* after the linked story, suggesting varied knowledge of **π** digits among the students coming into the class exercise. Furthermore, the results corroborate the relevance of historical small-*N* methodology for the study of cognitive processes that otherwise would be considered appropriately studied using group-design methodology in today’s psychological research world. Finally, by providing “real-world” ([70, 103]) extensions, they support the generality of these classic reports of memory phenomena from the standpoint of the second research tradition of psychology noted in the introduction. In that tradition:

Contrary to what is usually assumed about the small-*N* experimental approach, namely, that it lacks generality due to the sample size that is usually small compared to what is typical in the alternative group-design approaches, generality is of paramount interest and is usually accounted for in behavior analytic research. Replication is what affirms generality, especially of the type sought after by mainstream psychologists. ([18, 25, 27])

### Pedagogic and methodological implications and historical antecedents.

“Significance testing never makes a useful contribution to the development of cumulative science.” [33

In light of the ongoing replication crisis in psychology (e.g., [5, 6]), the results of this report are worthy of note for both pedagogic and research purposes. Pedagogically, they illustrate the value of such in-class activities in demonstrating psychological phenomena that have a firm foundation of empirical reproducibility, much like using physics demonstration experiments to illustrate established physical principles (e.g., [104]). Indeed, if they were not so firmly established, they would be deficient as activities for demonstrating psychological principles because they would be vicariously haphazard and unpredictable and therefore unworthy as classroom demonstrations. As Poling et al. [64] pointed out, “[*i]n science, repeatability is tantamount to believability*. Relations that can be reproduced are accepted as real; those that cannot be reproduced are rejected” ([64, 96] IM).

For research purposes, the history of the entrance and ascendance of inferential statistics into psychology is illuminating. The actual coupling of psychological research and statistical inference [58, 105] defined the path that separated mainstream psychology and behavior analysis [25] leaving the former with and the latter without a pervasive replication problem (see [18] for similar case made for vision research). As mentioned in the introduction, replications tend to be associated naturally with small-*N* designs [51, 63, 106]. According to Stigler, following Pierce’s adoption of “randomization to create an artificial baseline…, Fechner’s control of experimental conditions, like that of Muller, Wundt, and Ebbinghaus, created an artificial baseline and a framework that made statistical investigation possible. Psychology has never been the same since” [58, 61, 64]).

The issues and problems introduced by the wholesale embrace of NHST in psychology seem not to have been necessary for a productive scientific endeavor to create a cumulative science (see [39, 107]) prior to the coupling of inferential statistics and research in psychology. Hubbard and Ryan’s [88] findings on APA journals’ reporting of inferential statistics showed how empirical research before the 1910s in psychology did not rely on statistical inference to make sound decisions about psychological results. Boring’s [62] report of “experimental control” in the *American Journal of Psychology* followed a similar historical pattern, with increasing use of control groups or comparisons from the mid-1910s through the early 1950s following the rise of NHST. Indeed, most, if not all, discovery of foundational principles occurred under experiments conducted without statistical tools of the sorts in widespread use in psychological research today. One has to ask what the benefit is for introducing these tools: Are new discoveries better because of their introduction? Developments that retard progress are not worth having (see [2]). We should adopt and embrace tools because they make our march towards a cumulative science possible, not because they make doing the science convenient for us, as NHST does.

### Implications of small-*N* designs and visualization of effects

Others have noted the important role expert judgements play in doing science (see, e.g., [51, 108, 109]) and advocated for their use in psychological research [25]. Applying expert judgement may not be convenient and “quick” to the task of getting a manuscript out in a timely manner, but applying the dichotomous, yes or no, answer that NHST affords certainly is (see [110, 111]). When conditions change, such as when an elaborative-rehearsal task as opposed to a maintenance-rehearsal task, precedes the memory test of a previously encountered learning material, the perceptible difference in recall of the material can be visualized readily even without expertise. This was the case as the students did following the depth of processing demonstration, see Fig. 3 for the graphical shifts in items recalled in that exercise. Graphical visualization is a recommended best practice [19, 49], see also [112] at any rate, and its use in decision-making can be trained (e.g., [113–116]). Expertise in use of visual inspection thus is demonstrably trainable. Nevertheless, as the earliest generations of psychological researchers have amply demonstrated (see [88]), extant research practices do not have to involve inferential statistics to be valuable and productive. Although the replication crises arose in the context of use of NHST, indeed, many of these pre-1910 studies were not memory studies and yet they reported findings without inferential statistics. They, thus, precluded the possibility that non-memory psychological phenomena could not be studied and reported without inferential statistics like the classic memory studies replicated in the present study.

As noted in the introduction, Ebbinghaus’ study of memory was one of the important psychological reports that used *N* = 1 research [50]. Psychology’s early and later history is replete with such a research approach [71] that did not involve the use of significance testing at all. Classic discoveries in psychology other than the psychophysical ones mentioned above such as Fechner’s (e.g., see [54, 65]) that did not use a *t*- or *F*-test nor report any *p* values, or even confidence intervals or Bayes factors are numerous. Among the works so identified by Gigerenzer and Marewski are Jean Piaget’s child development stages (see, e.g., [117]), Wolfgang Köhler’s ape intelligence [118] and his Gestalt laws of perception [119], Ivan P. Pavlov’s principles of classical conditioning (see [120]), B. F. Skinner’s principles of operant conditioning (see [121]), George Miller’s magical 7 ± 2 (see [74]), and Herbert A. Simon’s Nobel Prize–winning work in economics. Over and above the “methodological eclecticism” in the pursuit of measurement precision that allowed Ebbinghaus to achieve such acclaim in the study of memory [95

All of these characteristics attest to the possibility of a psychological science conducted without the use of group design and/or NHST. Piaget, for example, reported hundreds of detailed vignettes of cases to illustrate, demonstrate, or support his theories of development without ever adopting an experimental design that involved groups of children [117, 122]. Despite his oppositions to the behaviorism of his day, largely on opposing views on the epistemological status of objective reality and personal experience arising from respective positions on introspection, Köhler was sympathetic to Watson’s use of qualitative observations of children and objected to what he called the “quantitative method” that required statistical analysis of data. As he retorted, “[e]verything that is valuable in these observations would disappear if ‘results’ were handled in an abstract statistical version” [119*N* experimental designs, which are distinct from the prevalent mainstream group designs and NHST.

### What is to become of psychology?

“A student can complete our graduate program without learning anything at all about basic learning processes, or basic sensory and perceptual processes, or memorial and cognitive processes, or basic developmental processes, or social processes, or approaches to personality, and so on. Students, as in most graduate programs, can pick and choose among a few courses on those (and other) topics to provide them presumed breadth. But the only training *every* student must have is in NHST… this state of affairs has developed because of the reliance on NHSTs as the dominant method for analyzing data and deciding if results merit publication, thus retarding the development of cumulative, evolving, integrated knowledge.” [39

“The experimental means for groups of adults generally range from about 3–5 chunks, whereas individual subject means range more widely from 2 to 6 chunks.” [97

Can psychology be defined as the study of *average* behavior and mental phenomena as opposed to the now standard, study of behavior and mental processes (e.g., [96])? An alien looking in could, indeed, surmise that psychology *is* the study of the *average person*, not of processes (c.f., [18, 51]), by the overwhelming reliance on group designs in contemporary psychological research, which continually yield reports of averages. Not all psychological phenomena are conducive to examination by group designs (in fact, many are not), however; just as human and nonhuman behavior tends to be an attribute of the individual, so are cognitions [18, 34]. Surely, there are behaviors and cognitions that manifest as group phenomena, but most things that psychologists are interested in tend to be those of the individual. This is true even of social psychology. Social psychologists do not study *average persons*, but social influences and perceptions as variables that affect individual behavior and/or cognition. Phenomena like groupthink may be exceptions, and even then, the unit of interest is the group, not an *average person*.

Perhaps the best way, going forward, in initiating a research project is, first, to determine primarily if the phenomenon of interest is an attribute of the individual or a group process and only then, second, to choose an appropriate design that fits the phenomenon. A behavior and/or cognition that is fundamentally of/about the individual is better studied with designs that appropriately answers questions about the individual and not about the average person or animal (it is possible, I guess, to be interested in the average person or animal per se, in which case the appropriate design of choice would be the group design). A recent report on altruism in rodents [123] is illustrative. There have been questions on whether rodents engage in prosocial behavior for empathetic or altruistic reasons (e.g., [124, 125]) or for social-contact reasons (e.g., [126]), a presumably social albeit biologic behavior. It took a systematic replication with small-*N* experimental design and reconfiguration of equipment and of the prevailing economy of the test environment to seek out the controlling variables in what appears to be a case of altruism *prima facie* (see [123]). Refocusing the question informed the methodology deployed, which yielded ostensibly greater scientific clarity.

Finally, even Sidman is on record for saying that actuarial and other social or policy matters may actually require the use of and reliance on statistics (see [51, 127]). It is therefore only a matter of perceptive choice of methodology tailored appropriately to a research question on the behavior and/or cognition of the individual or of the group. The works of Guilford and Dallenbach [73] and Oberly [75] on immediate memory span described above are illustrative in combining features of small-*N* (in their intensive parts) and large-*N* (in their extensive parts) in the same studies, even without the aid of inferential statistics to grasp the meaning and interpretation of their results. In pointing out that endorsements of small-*N* designs is not a one-size-fits-all proposition, Smith and Little made a case for accommodating both small- and large-*N*: “When the goal is to estimate population parameters,…then the recommendation to increase sample size *at the participant level* is an appropriate one” ([18, 95] and Colling and Szucs’ “*pragmatic pluralism*” in calling for the adoption of both frequentist and Bayesian inferential approaches in psychological research. According to Colling and Szucs, “statistical reform is necessary because it is necessary to have the right tool for the right job in a complete system of *scientific inference*” [21*N* design as was possible with memory (e.g., [73, 75]) in this case, for example, one simply adopts the appropriate design and the relevant statistical analyses. Such a methodological position is similar, at least in spirit, to Holtz’s [110] recommendations for epistemological solutions to the ongoing crises of confidence in psychology. As Smith and Little put it, “[i]n environments that can be explored at the individual level and the phenomenon of interest is expressed as an individual-level mechanism, small-*N* studies have enormous inferential power and are preferable to group-level inference precisely because they place the burden of sampling at the appropriate level, that of the individual” ([18, 27]).

## Conclusions

The ongoing crises of confidence in psychology have been attributed variously to a collection of related factors in the practice of our science. The attributions need not be of one-track solution focused mainly at research practices of only one of psychology’s long-established traditions, however. The results reported here are remarkable and noteworthy in validating these historically important psychological findings outside of the laboratory. They are testament to the reliability of those reproducible effects.

What we have today is a divided attention to inferential statistical considerations of only one of psychology’s research traditions, with outright neglect of the other well-nourished and empirically productive alternative. Rather, what is required is a more pragmatic approach of considered attention to the research question, to the selection of appropriate research design and analyses, and to informed theoretical framework in which to situate properly our understanding of the outcomes. This position is neutral to the question of whether psychology’s crises of confidence arose from the statistical tool-user or the tool itself, alluded to above, so long as the research question drives the informed choice of the design and the educated use of the relevant tools, statistical and otherwise. The choice of designs and the appropriate statistical and/or other tools are, of course, in the purview of expert judgement [25, 51] exercised by the researcher in his/her research domain.

## Data Availability

All data generated or analyzed in this study are included in this published article. The raw datasets used and analyzed during the study are available from the author on reasonable request.

## Abbreviations

QRPs: Questionable research practices
NHST: Null hypothesis statistical testing
LLN: Law of large numbers
SST: Statistical significance testing
SHT: Statistical hypothesis testing
APA: American Psychological Association
IM: Instructor’s materials
IMS: Immediate memory span
DoP: Depth of processing

## References

[1] Hanin L (2021). Cavalier use of inferential statistics is a major source of false and irreproducible scientific findings. Mathematics.

[2] Ioannidis JPA (2012). Why science is not necessarily self-correcting. Perspect Psychol Sci.

[3] Chung S, Fink EL (2018). One of the most cited persuasion studies but no success in replication: investigating replication using Petty, Cacioppo, and Goldman (1981) as an example. Ann Int Commun Assoc.

[4] Bem DJ (2011). Feeling the future: experimental evidence for anomalous retroactive influences on cognition and affect. J Personal Soc Psychol.

[5] Ritchie SJ, Wiseman R, French CC (2012). Failing the future: three unsuccessful attempts to replicate Bem’s retroactive facilitation of recall effect. PLoS ONE.

[6] Cesario J (2014). Priming, replication, and the hardest science. Perspect Psychol Sci.

[7] Ferguson MJ, Carter TJ, Hassin RR (2014). Commentary on the attempt to replicate the effect of the American flag on increased Republican attitudes. Soc Psychol.

[8] Klatzky RL, Creswell JD (2014). An intersensory interaction account of priming effects—and their absence. Perspect Psychol Sci.

[9] Klein RA, Ratliff KA, Vianello M, Adams RB, Bahnik S, Bernstein MJ, Nosek BA (2014). Investigating variation in replication: a “many labs” replication project. Soc Psychol.

[10] Spellman BA (2015). A short (personal) future history of revolution 2.0. Perspect Psychol Sci.

[11] Holland SM (2019). Estimation, not significance. Paleobiology.

[12] McManus E, Turner D, Sach T (2019). Can you repeat that? Exploring the definition of a successful model replication in health economics. PharmcoEconomics.

[13] Roloff J, Zyphur MJ (2019). Null findings, replication and preregistered studies in business ethics research. J Bus Ethics.

[14] Wohl MJA, Tabri N, Zelenski JM (2019). The need for open science practices and well-conducted replications in the field of gambling studies. Int Gamb Stud.

[15] Vermeuhen I, Beukeboom CJ, Batenburg A, Avramiea A, Stoyanov D, van de Velde B, Oegema D (2015). Blinded by the light: how a focus on statistical ‘significance’ may cause p-value misreporting and an excess of p-values just below.05 in communication science. Commun Methods Meas.

[16] Simmons JP, Nelson LD, Simonsohn U (2011). False-positive psychology: undisclosed flexibility in data collection and analysis allows presenting anything as significant. Psychol Sci.

[17] Little DR, Smith PL (2018). Replication is already mainstream: lessons from small-*N* designs. Behav Brain Sci.

[18] Smith PL, Little DR (2018). Small is beautiful: in defense of the small-*N* design. Psychon Bull Rev.

[19] Cumming G (2014). The new statistic: Why and how?. Psychol Sci.

[20] Pashler H, Wagenmakers E (2012). Special section on replicability in psychological science: A crisis of confidence?. Perspect Psychol Sci.

[21] Colling LJ, Szucs D (2021). Statistical inference and the replication crisis. Rev Philos Psychol.

[22] Cumming G, Fidler F (2009). Confidence intervals: better answers to better questions. J Psychol.

[23] Kruschke JK, Liddell TM (2018). The Bayesian new statistics: hypothesis testing, estimation, meta-analysis, and power analysis from a Bayesian perspective. Psychon Bull Rev.

[24] Wagenmakers E-J (2007). A practical solution to the pervasive problems of *p* values. Psychon Bull Rev.

[25] Imam AA (2021). Historically recontextualizing Sidman’s *Tactics*: how behavior analysis avoided psychology’s methodological Ouroboros. J Exp Anal Behav.

[26] Hurtado-Parrado C, Lopez-Lopez W (2015). Single-case research methods: history and suitability for a psychological science in need of alternatives. Integr Psychol Behav Sci.

[27] Normand MP (2016). Less is more: psychologists can learn more by studying fewer people. Front Psychol.

[28] Falk R, Greenbaum CW (1995). Significance tests die hard: the amazing persistence of a probabilistic misconception. Theory Philos.

[29] Ioannidis JPA (2005). Why most published research findings are false. PLoS Med.

[30] Morrison DE, Henkel RE (1070). The significance test controversy: a reader.

[31] Nickerson RS (2000). Null hypothesis significance testing: a review of an old and continuing controversy. Psychol Methods.

[32] Rozeboom WW (1960). The fallacy of null hypothesis significance test. Psychol Bull.

[33] Schmidt FL, Hunter JE, Harlow LL, Mulaik SA, Steiger JH (1997). Eight common but false objections to the discontinuation of significance testing in the analysis of research data. What if there were no significance tests?.

[34] Grice J, Barrett P, Cota L, Felix C, Taylor Z, Garner S, Medellin E, Vest A (2017). Four bad habits of modern psychologists. Behav Sci.

[35] Imam AA, Frate M (2019). A snapshot look at replication and statistical reporting practices in psychology journals. Eur J Behav Anal.

[36] Schneider JW (2015). Null hypothesis significance tests. A mix-up of two different theories: the basis for widespread confusion and numerous misinterpretations. Scientometrics.

[37] Lambdin C (2012). Significance tests as sorcery: science is empirical—significant tests are not. Theory Psychol.

[38] Bernard C. An introduction to the study of experimental medicine. Dover Publications Inc; (1927/1957).

[39] Branch M (2014). Malignant side effects of null-hypothesis significance testing. Theory Psychol.

[40] Harlow LL, Mulaik SA, Steiger JH (1997). What if there were no significance tests?.

[41] Gandevia S, Cumming C, Amrhein V, Butler A (2021). Replication: do not trust your p-value, be it small or large. J Physiol.

[42] Spellman BA (2012). Special section on research practices. Perspect Psychol Sci.

[43] Barry AE, Valdez D, Goodson P, Szucs L, Reyes JV (2019). Moving college health research: reconsidering our reliance on statistical significance testing. J Am Coll Health.

[44] Estes WK (1997). On the communication of information by displays of standard errors and confidence intervals. Psychon Bull Rev.

[45] Schmidt FL, Hunter JE (2002). Are there benefits from NHST?. Am Psychol.

[46] Tryon WW (2016). Replication is about effect size: comment on Maxwell, Lau, and Howard (2015). Am Psychol.

[47] Watson JC, Lenz AS, Schmit MK, Schmit EL (2016). Calculating and reporting estimates of effect size in counseling outcomes research. Couns Outcome Res Eval.

[48] Dienes Z (2015). How Bayes factors change scientific practice. J Math Psychol.

[49] American Psychological Association (2020). Publication manual of the American Psychological Association: the official guide to APA style.

[50] Dukes WF (1965). *N* = 1. Psychol Bull.

[51] Sidman M. Tactics of scientific research: evaluating experimental data in psychology. Authors Cooperative; 1960.

[52] Harrison JM, Turnock MT (1975). Animal psychophysics: improvements in the tracking method. J Exp Anal Behav.

[53] Krantz JH. Psychophysics. In: Experiencing sensation and perception (Chapter 2) (n.d.). https://psych.hanover.edu/classes/sensation/chapters/Chapter%202.pdf.

[54] Krantz JH, Davis SF, Buskist W (2008). Psychophysics. 21st Century psychology: a reference handbook.

[55] Read JCA (2015). The place of human psychophysics in modern neuroscience. Neuroscience.

[56] White KG, Wixted JT (1999). Psychophysics of remembering. J Exp Anal Behav.

[57] Blakemore C, Sutton P. Size adaptation: a new aftereffect. Science. 1969;166:245–247. 10.1126/science.166.3902.245 5809598

[58] Stigler SM (1992). A historical view of statistical concepts in psychology and educational research. Am J Educ.

[59] Branch M (1999). Statistical inference in behavior analysis: some things significance testing does and does not do. Behav Anal.

[60] Perone M (1999). Statistical inference in behavior analysis: experimental control is better. Behav Anal.

[61] Saville BK, Davis SF, Buskist W (2008). Single-subject designs. 21st Century psychology: a reference handbook.

[62] Boring EG (1954). The nature and history of experimental control. Am J Psychol.

[63] Branch M (2021). Lessons worth repeating: Sidman’s Tactics of Scientific Research. J Exp Anal Behav.

[64] Poling A, Methot LL, LeSage MG (1995). Fundamentals of behavior analytic research.

[65] Boring EG (1961). The beginning and growth of measurement in psychology. Isis.

[66] Catania AC (2007). Learning.

[67] Bachelder BL, Delprato DJ (2017). The simple memory span experiment: a behavioral analysis. Psychol Rec.

[68] Ferguson CJ (2015). “Everyone knows psychology is not a real science”: public perceptions of psychology and how we can improve our relationship with policymakers, the scientific community, and the general public. Am Psychol.

[69] Francis G (2012). Publication bias and the failure of replication in experimental psychology. Psychon Bull Rev.

[70] Huffmeier J, Mazei J, Schultze T (2016). Reconceptualizing replication as a sequence of different studies: a replication typology. J Exp Soc Psychol.

[71] Gigerenzer G, Marewski JN (2015). Surrogate science: the idol of a universal method for scientific inference. J Manag.

[72] Laws KR (2016). Psychology, replication and beyond. BMC Psychology.

[73] Guilford P, Dallenbach KM (1925). The determination of memory span by the method of constant stimuli. Am J Psychol.

[74] Miller GA (1956). The magical number seven, plus or minus two: some limits on our capacity for processing information. Psychol Rev.

[75] Oberly HS (1928). A comparison of the span of attention and memory. Am J Psychol.

[76] Murray DJ (1976). Research on human memory in the nineteenth century. Can J Psychol Rev Can Psychol.

[77] Craik FIM, Lockhart RS (1972). Levels of processing: a framework for memory research. J Verb Learn Verb Behav.

[78] Pollack I (1953). Assimilation of sequentially encoded information. Am J Psychol.

[79] Carmichael L, Hogan HP, Walter AA (1932). An experimental study of the effect of language on the reproduction of visually perceived form. J Exp Psychol.

[80] Munsterberg H (1894). Studies from the Harvard psychological laboratory (I): memory. Psychol Rev.

[81] Henmon VAC (1917). The relation between learning and retention and amount to be learned. J Exp Psychol.

[82] Luh CW (1922). The conditions of retention. Psychol Monogr.

[83] Mibai S (1922). The effects of repetitions upon retention. J Exp Psychol.

[84] Sauer FM (1930). The relative variability of nonsense syllables and words. J Exp Psychol.

[85] Murre JMJ, Dros J (2015). Replication and analysis of Ebbinghaus’ forgetting curve. PLoS ONE.

[86] Tulving E (1985). Ebbinghaus’s memory: What did he learn and remember?. J Exp Psychol Learn Mem Cognit.

[87] Kirkpatrick EA (1894). An experimental study of memory. Psychol Rev.

[88] Hubbard R, Ryan PA (2000). The historical growth of statistical significance testing in psychology—and its future prospects. Educ Psychol Meas.

[89] Moscovitch M, Craik FIM (1976). Depth of processing, retrieval cues, and uniqueness of encoding as factors in recall. J Verb Learn Verb Behav.

[90] Bobrow SA, Bower GH (1969). Comprehension and recall of sentences. J Exp Psychol.

[91] Hyde TS, Jenkins JJ (1969). The differential effects of incidental tasks on the organization of recall of a list of highly associated words. J Exp Psychol.

[92] Johnston CD, Jenkins JJ (1971). Two more incidental tasks that differentially affect associative clustering in recall. J Exp Psychol.

[93] Rosenberg S, Schiller WJ (1971). Sematic coding and incidental sentence recall. J Exp Psychol.

[94] Tresselt ME, Mayzner MS (1960). A study of incidental learning. J Psychol.

[95] Postman L (1968). Hermann Ebbinghaus. Am Psychol.

[96] Bernstein DA (2010). Essentials of psychology.

[97] Cowan N (2000). The magical number 4 in short-term memory: a reconsideration of mental storage capacity. Behav Brain Sci.

[98] Cowan N (2000). Metatheory of storage capacity limits. Behav Brain Sci.

[99] Bachelder BL (2000). The magical number 4 = 7: span theory on capacity limitations. Behav Brain Sci.

[100] Baddeley A (2000). The magic number and the episodic buffer. Behav Brain Sci.

[101] Kawai N, Matsuzawa T (2000). “Magical number 5” in a chimpanzee. Behav Brain Sci.

[102] Towse JN (2000). Memory limits: “Give us an answer!”. Behav Brain Sci.

[103] Gantman A, Gomila R, Martinez JE, Matias EN, Paluck EL, Starck J, Wu S, Yaffe N (2018). A pragmatist philosophy of psychological science and its implications for replication. Behav Brain Sci.

[104] Stewart SM. Some physics demonstration experiments. Science Papers. 2005, pp 121–133. https://www.researchgate.net/publication/256120711_Some_simple_physics_demonstration_experiments.

[105] Cowles M (2001). Statistics in psychology: an historical perspective.

[106] Lemon CJ, King SA, Davidson KA, Berryessa TL, Gajjar SA, Sacks LH (2016). An inadvertent concurrent replication: same roadmap, different journey. Remed Spec Educ.

[107] Meehl PE (1978). Theoretical risks and tabular asterisks: Sir Karl, Sir Ronald, and slow progress of soft psychology. J Consult Clin Psychol.

[108] Cohen J (1990). Things I have learned (so far). Am Psychol.

[109] Davidson IJ (2018). The Ouroboros of psychological methodology: the case of effect sizes (Mechanical objectivity vs. expertise). Rev Gen Psychol.

[110] Holtz P (2020). Two questions to foster critical thinking in the field of psychology: Are there any reasons to expect a different outcome, and what are the consequences if we don’t find what we were looking for?. Meta-Psychology.

[111] Russell MK, Hall MD (2019). Responding to confidence and reproducibility crises: registered reports and replication in auditory perception and cognition. Audit Percept Cognit.

[112] Levine SS (2018). Show us your data: connect the dots, improve science. Manag Organ Rev.

[113] Kipfmiller KJ, Brodhead MT, Wolfe K, LaLonde K, Sipila ES, Bak MYS, Fisher MH (2019). Training frontline employees to conduct visual analysis using a clinical decision-making model. J Behav Educ.

[114] Ninci J, Vannest KJ, Willson V, Zhang N (2015). Interrater agreement between visual analysts of single-case data: a meta-analysis. Behav Modif.

[115] Retzlaff BJ, Phillips LA, Fisher WW, Hardee AM, Fuhrman AM (2020). Using e-learning modules to teach ongoing-visual inspection of functional analysis. J Appl Behav Anal.

[116] Wolfe K, McCammon MN, LeJeune LM, Holt AK (2021). Training preservice practitioners to make data-based instructional decisions. J Behav Educ.

[117] Piaget J. The construction of reality in the child. Cook, M, translator. Basic Books; 1954

[118] Köhler W (1925). The mentality of apes.

[119] Köhler W (1947). Gestalt psychology: an introduction to new concepts in psychology.

[120] Pavlov IP. Conditioned reflexes. Dover Publications; 1927/1960.

[121] Skinner BF (1938). The behavior of organisms: an experimental analysis.

[122] Piaget J, Inhelder B, Szeminska A (1960). The child’s conception of geometry.

[123] Wan H, Kirkman C, Jensen G, Hackenberg TD (2021). Failure to find altruistic food sharing in rats. Front Psychol.

[124] Ben-Ami Bartal I, Decety J, Mason P (2011). Empathy and pro-social behavior in rats. Science.

[125] Sato N, Tan L, Tate K, Okada M (2015). Rats demonstrate helping behavior toward a soaked conspecific. Anim Cognit.

[126] Hachiga Y, Schwartz LP, Silberberg A, Kearns DN, Gomez M, Slotnick B (2018). Does a rat free a trapped rat due to empathy or for sociality?. J Exp Anal of Behav.

[127] Iversen IH (2021). Sidman or statistics?. J Exp Anal Behav.

